# Do Triplets Have Enough Information to Construct the Multi-Labeled Phylogenetic Tree?

**DOI:** 10.1371/journal.pone.0103622

**Published:** 2014-07-31

**Authors:** Reza Hassanzadeh, Changiz Eslahchi, Wing-Kin Sung

**Affiliations:** 1 Department of Mathematics, Shahid Beheshti University, G.C., Tehran, Iran; 2 Department of Computer Science, Shahid Beheshti University, G.C., Tehran, Iran; 3 School of Computing, National University of Singapore, Singapore, Singapore; 4 Genome Institute of Singapore, Singapore, Singapore; Bangladesh University of Engineering and Technology, Bangladesh

## Abstract

The evolutionary history of certain species such as polyploids are modeled by a generalization of phylogenetic trees called multi-labeled phylogenetic trees, or MUL trees for short. One problem that relates to inferring a MUL tree is how to construct the smallest possible MUL tree that is consistent with a given set of rooted triplets, or SMRT problem for short. This problem is NP-hard. There is one algorithm for the SMRT problem which is exact and runs in 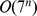 time, where 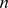 is the number of taxa. In this paper, we show that the SMRT does not seem to be an appropriate solution from the biological point of view. Indeed, we present a heuristic algorithm named MTRT for this problem and execute it on some real and simulated datasets. The results of MTRT show that triplets alone cannot provide enough information to infer the true MUL tree. So, it is inappropriate to infer a MUL tree using triplet information alone and considering the minimum number of duplications. Finally, we introduce some new problems which are more suitable from the biological point of view.

## Introduction

MUL trees are rooted phylogenetic trees where some leaves are labeled by the same taxa. They find applications in the study of the evolution of polyploids. The other applications of MUL trees include molecular systematics, biogeography, the study of host-parasite cospeciation and computer science [8], [11], [15], [18]–[20], [22]. In this paper we focus on rooted binary MUL trees. Several algorithms for constructing MUL trees from various datasets are introduced. Examples include building consensus MUL trees [6], [14], [15], constructing a phylogenetic network from a MUL tree [10] and transforming a collection of MUL trees into a collection of evolutionary trees [23]. One of the problems in the field of inferring MUL trees is to construct a smallest possible MUL tree consistent with a given set of rooted triplets, or SMRT problem for short. It is proved that SMRT is an NP-hard problem [9]. Up to now, a number of algorithms for inferring a phylogenetic tree or network from a set of triplets are presented [1], [4], [12], [13], [24]–[26]. However, there is only one algorithm for constructing a smallest possible MUL tree from a set of triplets [9]. This algorithm is exact and runs in 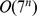 time where 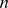 is the number of taxa. Here, we present the MTRT algorithm which is a heuristic method for the SMRT problem. MTRT is based on Aho et al.'s algorithm presented in [1]. Aho et al.'s algorithm is a top-down algorithm that constructs a rooted tree consistent with a given set of triplets, if such a tree exists. In the MTRT algorithm, we modify the Aho et al.'s algorithm to construct a MUL tree with the minimum number of duplications that is consistent with a given set of triplets. The duplication in a MUL tree is defined in the next section. We tested the performance of the MTRT algorithm on more than 400 biological and simulated datasets and showed that MTRT is efficient and can often find the optimal answer in practice. Furthermore, we showed that minimizing the number of duplications may not be an appropriate criterion for inferring a MUL tree.

### Preliminaries

A rooted triplet, or triplet for short, is a binary rooted tree on three distinct taxa. A triplet on three taxa 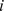, 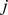 and 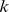 is denoted by 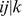 if the lowest common ancestor of 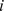 and 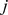 is a proper descendant of that of 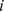 and 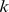, or 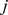 and 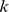. Let 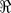 be a set of triplets on a taxa set 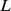. For any subset 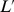 of 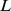, the set of all triplets 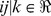 for which 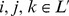 is called the set of triplets induced by 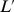 and is denoted by 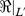. We also set 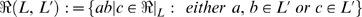. A triplet 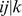 and a MUL tree 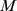 are said to be consistent if 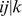 is an embedded subtree of 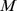. We say that a MUL tree 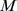 and a given set 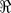 of rooted triplets are consistent if every triplet in 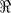 is consistent with 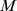. The set 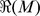 of all triplets consistent with 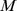 is called the triplet encoding of 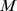. The following definitions are taken from [9]:

For any MUL tree 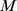, denote the set of all leaf labels that occur in 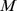 by 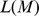. For any leaf label 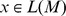, the number of duplications of 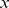 is equal to the number of occurrences of 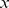 in 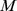 minus 1. The number of leaf duplications in 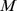, denoted by 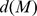, is the total number of duplications of all leaf labels in 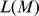. Define 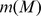 as the number of leaves in 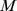. Then, 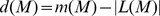. Now, we consider the following problem, called the smallest MUL tree from rooted triplets problem, or SMRT for short:

#### SMRT problem

Given a set 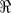 of rooted triplets over a leaf label set 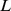, output a MUL tree 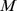 with 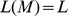 which is consistent with 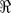 and minimizes 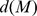.

## Results

### Simulation data

In this section, we report the results of our simulation study. For all data, the MTRT algorithm was run on a laptop with a 1.8 GHz Dual Core processor and 1GB RAM. MTRT is implemented in MATLAB. To test the performance of the algorithm, we simulated 400 MUL trees by 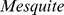 program [16]. This program can simulate and analyze gene trees from multiple populations. Three components must be established in Mesquite to do this:

1. A block of taxa representing the gene sequences.
2. A block of taxa representing the species (or populations).
3. A taxa association block, which is a special block of information that indicates how the taxa representing genes are associated with the taxa representing species.

Once these three components are established, Mesquite simulates gene trees by a coalescent process. The simulation starts at each extant population. Within each, the ancestry of the gene copies contained (as specified by the Taxa Association) is simulated by coalescence, going backward in time until the simulation arrives at the previous population (species) divergence. Mesquite makes this reconstruction under one assumption: that the only process occurring is gene duplication or extinction. Thus, the reconstruction reconciles the gene tree into the population tree so as to minimize the depths of gene tree divergences, which also minimizes gene duplication or extinction events, see [16] for more details.

**Figure 1:**
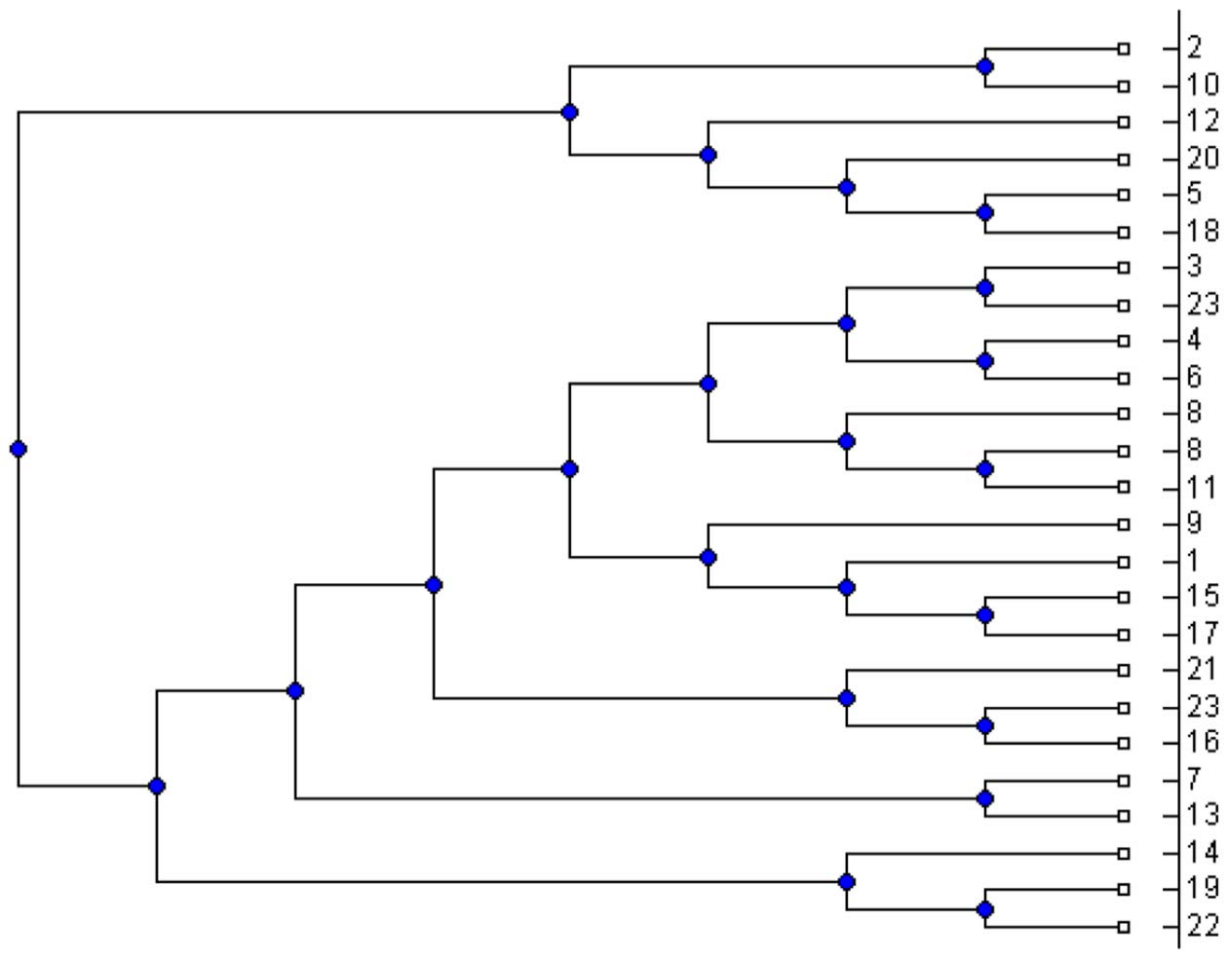
An original MUL tree used to test the MTRT algorithm.

**Figure 2:**
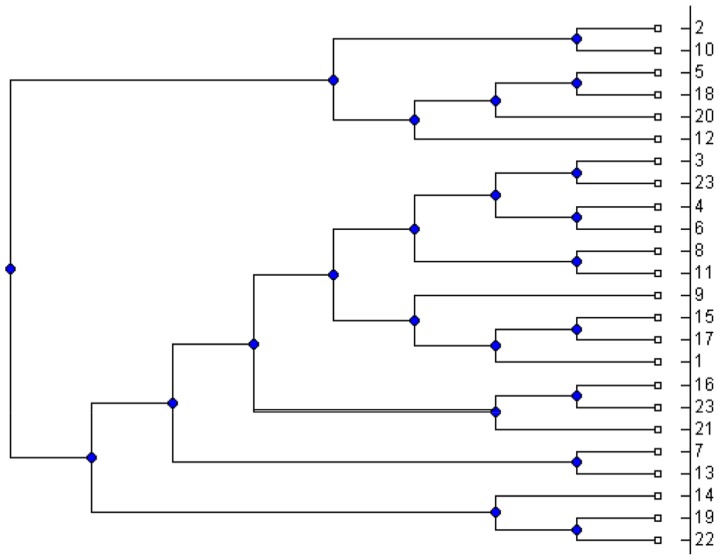
The obtained MUL tree by applying MTRT on the triplets extracted from the MUL tree shown in **Figure 1**.

Now we describe the procedure of simulating MUL trees. Suppose the gene tree 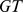 produced by Mesquite has 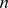 taxa. We considered the number of taxa for the species tree 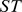 associated with 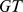 between 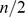 and 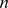. Then, we randomly indicated how the taxa representing genes are associated with the taxa representing species to obtain a taxa association block. After the simulation of the gene tree, to obtain a MUL tree, we replaced each gene by the species that belong to it. In all simulations, we considered 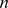 between 5 and 50. For each simulated MUL tree, we extracted all its triplets and applied the MTRT algorithm on the triplet set. The results show that in 42 percent of the datasets, MTRT produces a MUL tree which has less number of duplications than that of the original MUL tree. In only 10 percent of the datasets, the number of duplications for the output MUL tree of MTRT is greater than that of the original MUL tree. For the remaining 48 percent, the number of duplications for both MUL trees are the same. Hence, in 90 percent of the datasets, the algorithm MTRT constructs a MUL tree that has less or equal number of duplications than that of the original MUL tree. The minimum, maximum and average running times of the algorithm on 400 simulation datasets are 0.017, 40.36 and 9.1 seconds respectively. Figure 1 shows a simulated MUL tree. The output of the MTRT for the triplet set extracted from this MUL tree is given in Figure 2. The output MUL tree has one duplication while the original MUL tree has two duplications. We also compare MTRT with the exact algorithm presented in [9]. Since the exact algorithm requires exponential time and space, we can only run this algorithm on 100 small datasets which have 5–10 taxa. In 86 datasets, the MUL trees produced by both MTRT and exact algorithm have the same duplications. This shows that MTRT in many cases produces the smallest MUL trees for the triplet sets. For further study, we analysed the results of the exact algorithm. We found that, in 56 datasets, the exact algorithm produces a MUL tree which has less number of duplications than that of the original MUL tree.

### Real data

**Figure 3:**
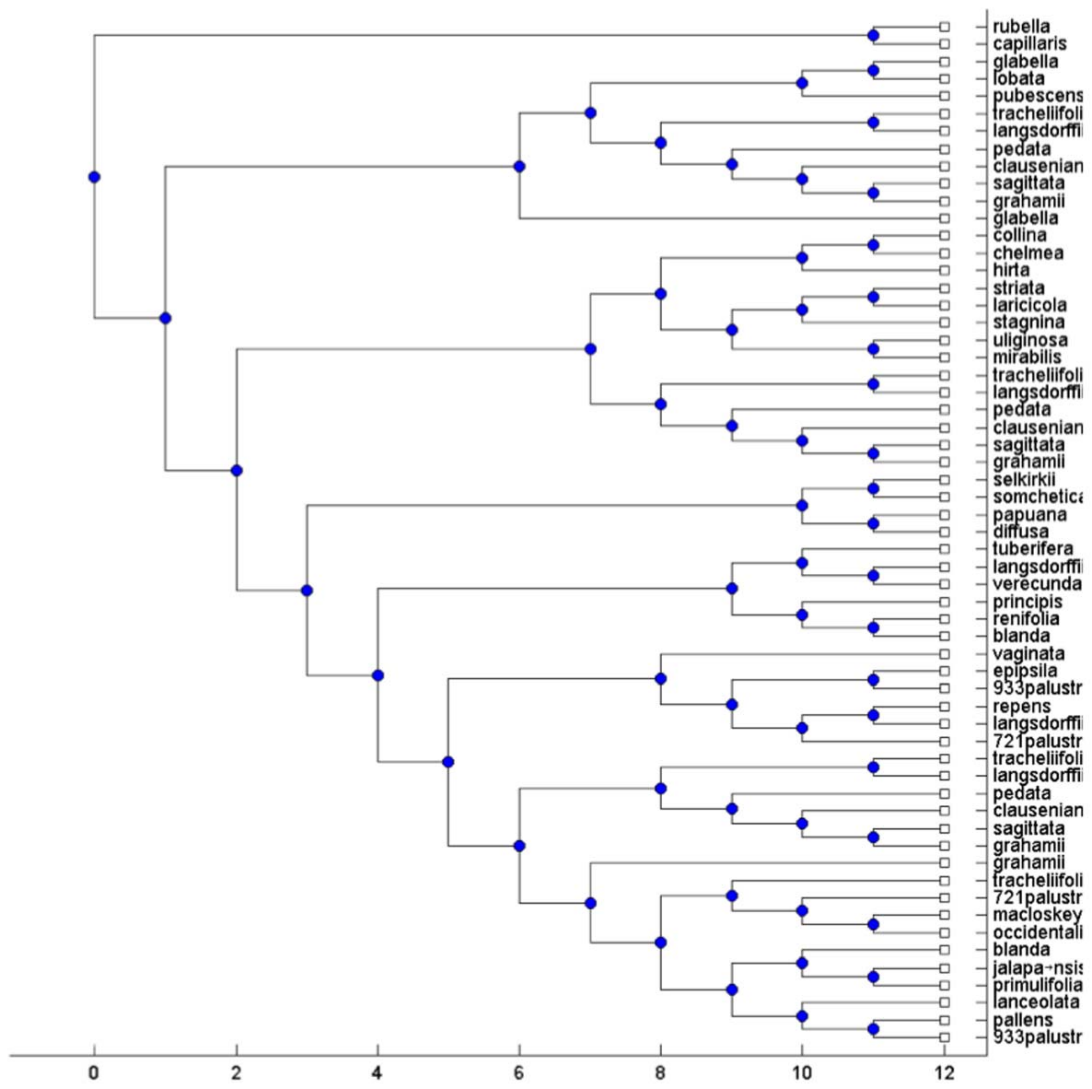
An original MUL tree on violet species with 20 duplications.

**Figure 4:**
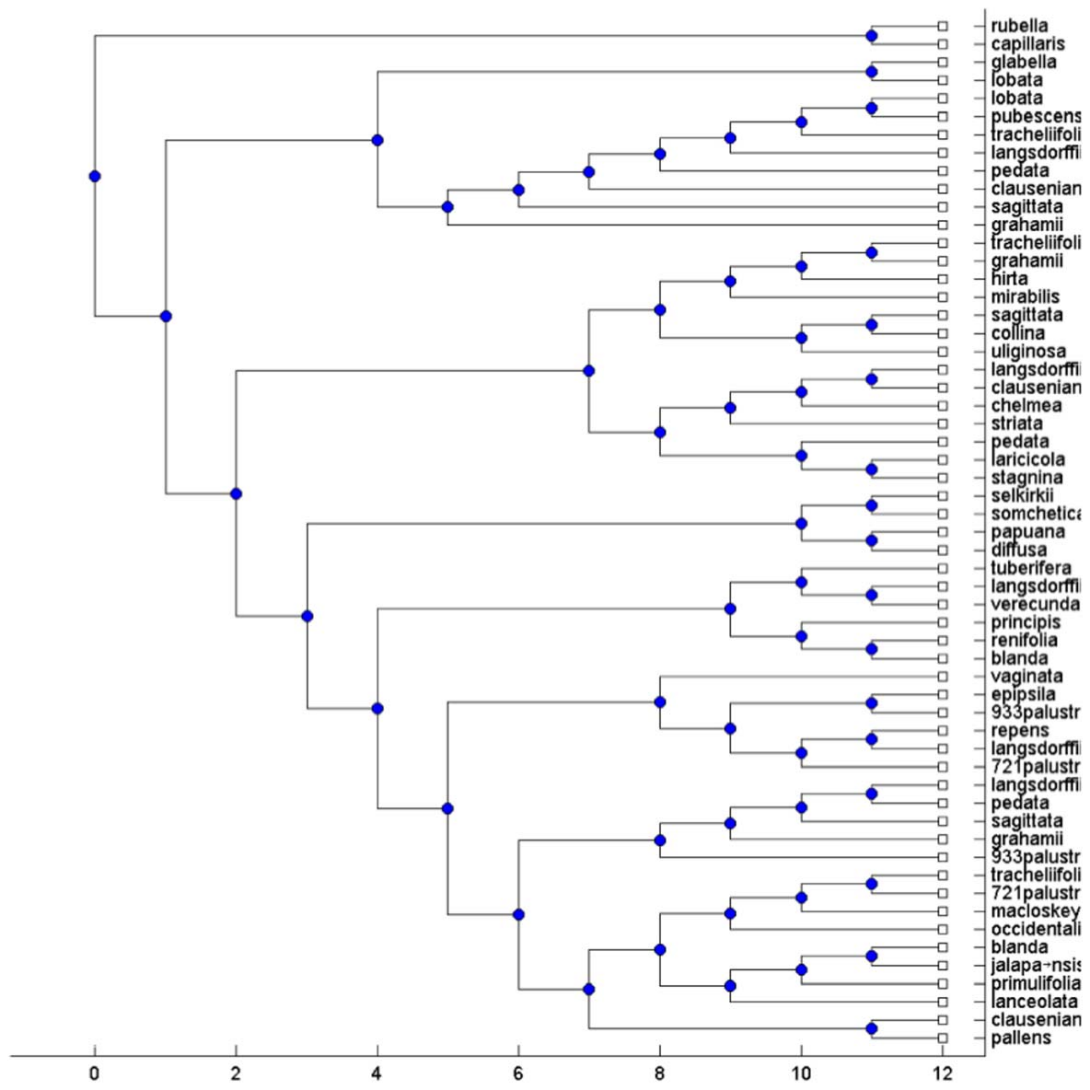
The obtained MUL tree by applying MTRT on the triplets extracted from the MUL tree shown in **Figure 3**. This MUL tree has 18 duplications.

**Figure 5:**
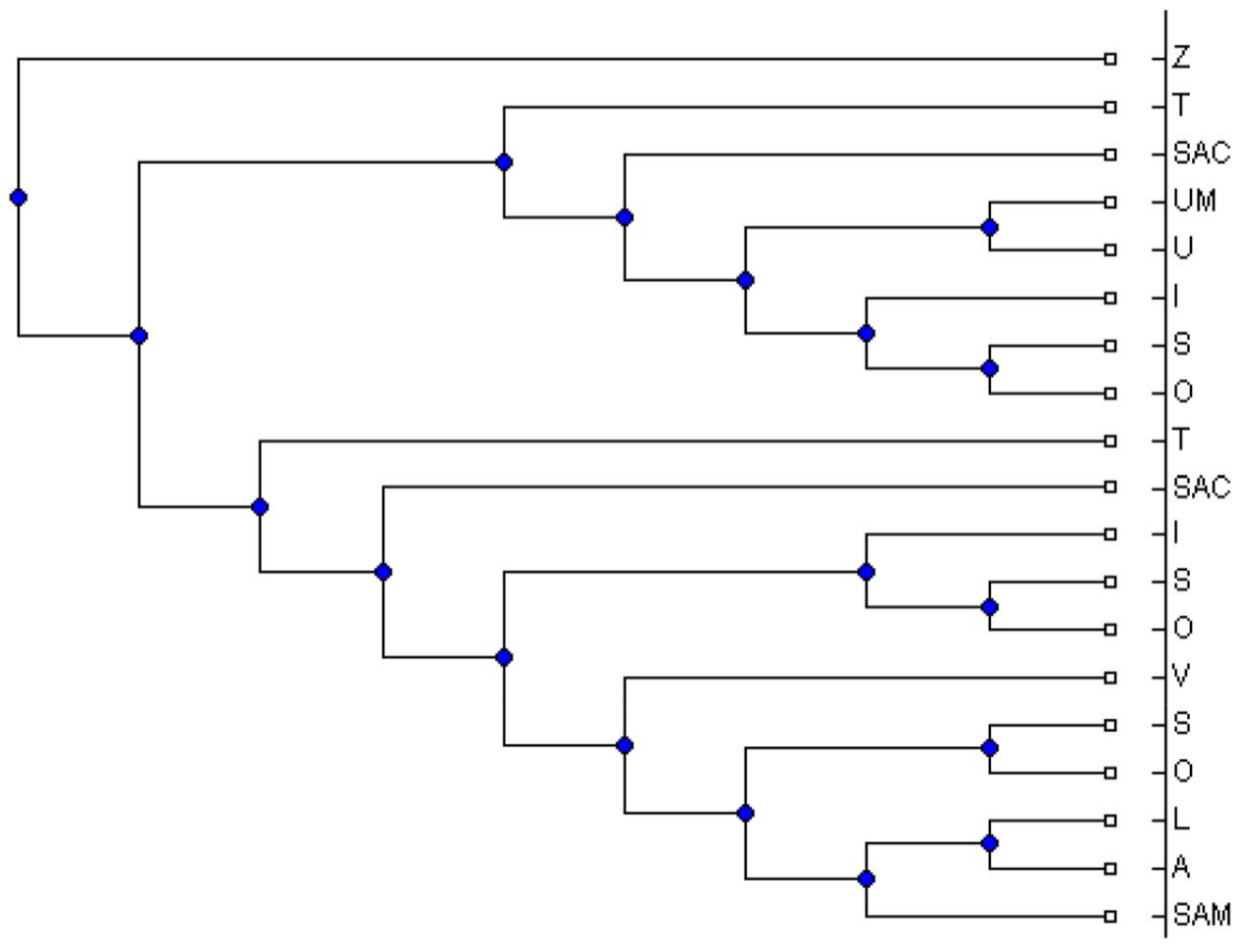
An original MUL tree on flowering plants with 7 duplications.

**Figure 6:**
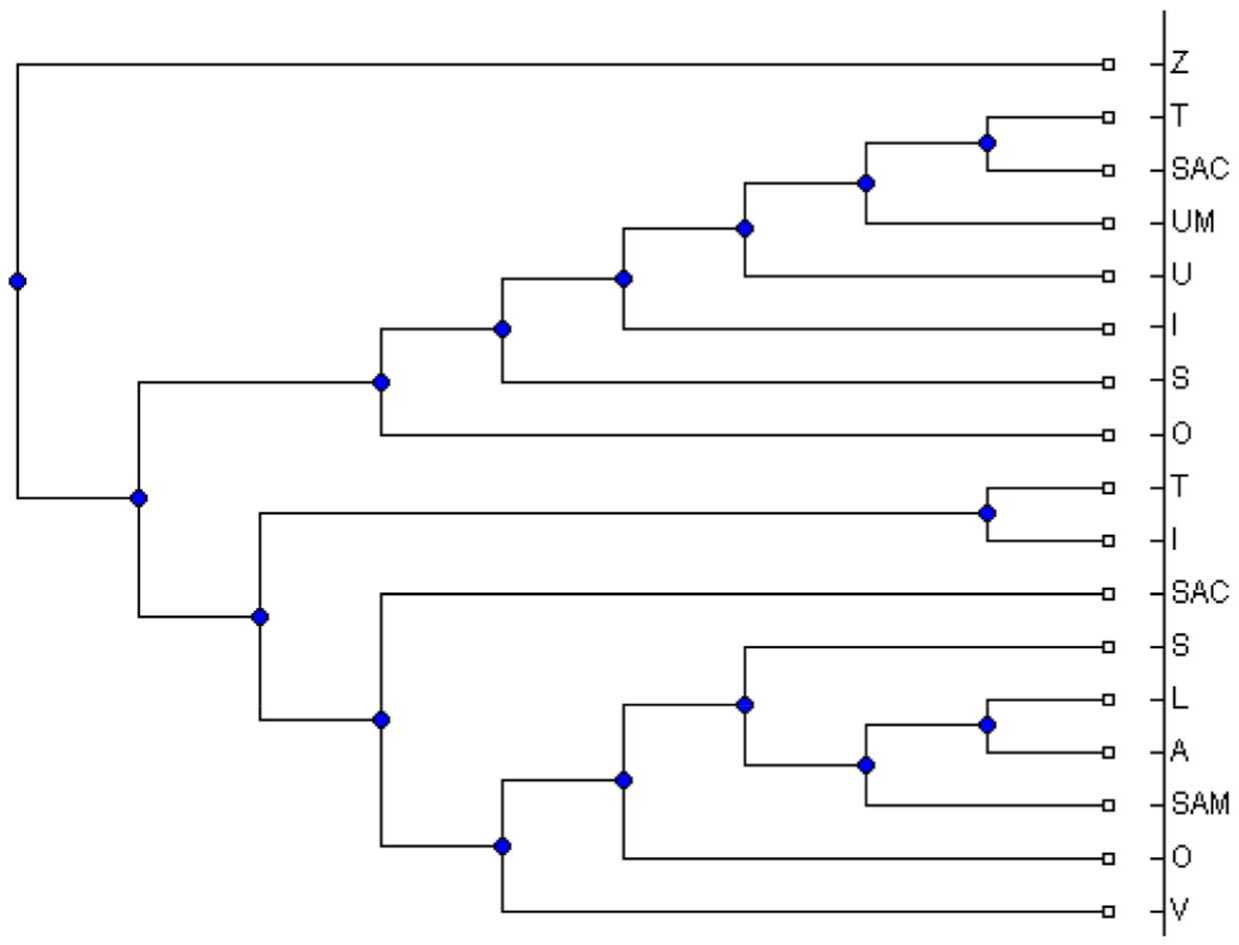
The obtained MUL tree by applying MTRT on the triplets extracted from the MUL tree shown in **Figure 5**. This MUL tree has 5 duplications.

To test the performance of the MTRT on real biological datasets, we applied MTRT on three datasets. The first and second datasets containing high-polyploid North American and Hawaiian violets [17]. All major morphological groups occurring in North America were sampled. All sequence were aligned with MUSCLE [7] and phylogenies were constructed using maximum likelihood. The third dataset containing the flowering plant genus Silene (Caryophyllaceae) was published in [21]. The gene trees in [21] are reconstructed using standard techniques in phylogenetic analysis from regions of the nuclear RNA polymerase gene family, two concatenated chloroplast regions and one nuclear ribosomal region, see [10] for more details. For each original MUL tree, we extracted all triplets and then apply MTRT on these triplets. In all cases, MTRT constructs a MUL tree which has less number of duplications than that of the original MUL tree. The original MUL trees for first and second datasets have 13 and 20 duplications, whereas the MUL trees produced by MTRT have 11 and 18 duplications respectively. Due to limitations of space, the MUL trees associated with one of the data are shown. Figure 3 and Figure 4 show the original MUL tree and the MUL tree constructed by MTRT for the triplet set extracted from the original MUL tree respectively. The original MUL tree for third dataset has 7 duplications, whereas the MUL tree produced by MTRT has 5 duplications. Figure 5 and Figure 6 show the original MUL tree and the MUL tree constructed by MTRT respectively. The labels represent Silene species, namely, S. ajanensis (A), S. uralensis (U), S. involucrata (I), S. sorensenis (S), S. ostenfeldii (O), S. zawadskii (Z), S. linnaeana (L), S. uralensis (Mongolia) (UM), S. samojedora (SAM), S. villosula (V), S. sachalinensis (SAC) and S. tolmatchevii (T).

### Reconstruction accuracy

For a phylogeny reconstruction algorithm, if a certain tree or network is used to obtain the input data, the algorithm should return exactly this tree or network. This is an important property for reconstructing phylogenies and known as the consistency principle. In the previous section, we observed that, for half of the simulated datasets and two real datasets, the number of duplications for input and output MUL trees are different. Further investigation showed that although some output MUL trees differ from input MUL trees, the outputs are consistent with all triplets corresponding to input MUL trees. In addition, we observed that some output MUL trees have more triplets than the corresponding input MUL trees. These observations show that inferring a MUL tree by minimizing the number of duplications may not properly detect biological properties and evolutionary relationships. So, there is a deficiency in the SMRT problem from a biological point of view. For further analysis, we used a concept which has already been defined for a tree called the rooted triplet distance to compare the output MUL trees with the input MUL trees [5].

#### Definition 1

**Figure 7:**
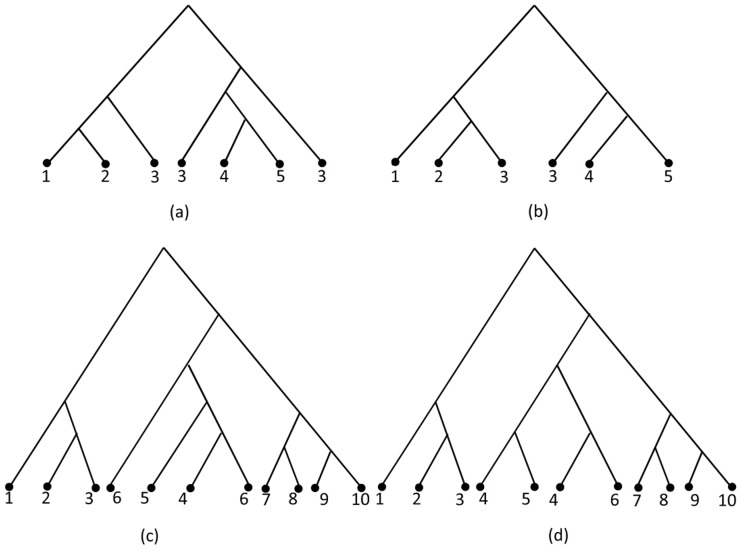
Comparing MUL trees using triplet distance. (a) The MUL tree 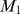, (b) The MUL tree 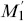 is consistent with 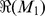. The MUL tree 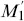 has less duplication than 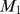 and is consistent with the triplet 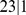 which is not contained in 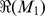. So, 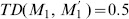, (c) The MUL tree 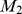, (d) The MUL tree 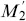 is consistent with 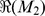. The MUL trees 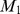 and 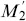 have the same number of duplications and 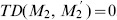.

**Figure 8:**
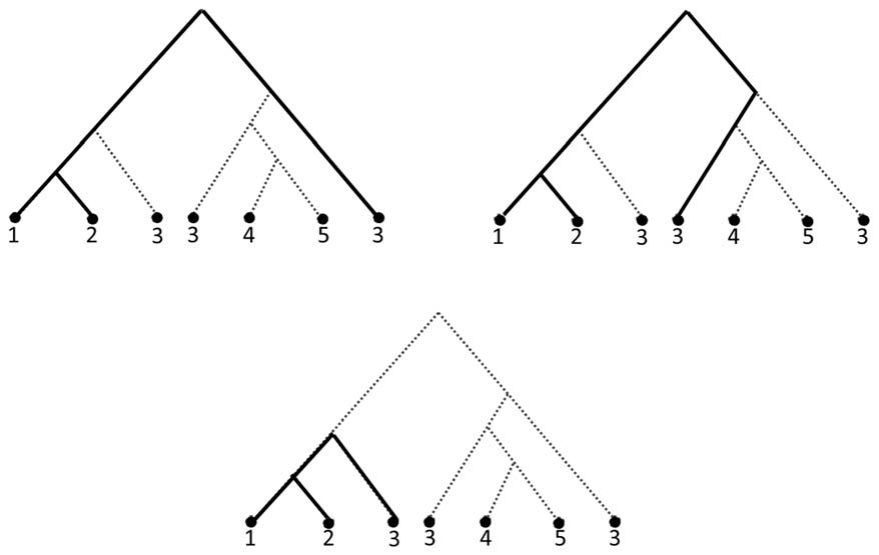
A MUL tree which has three different triplets 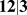.

The rooted triplet distance between two rooted phylogenetic trees 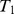 and 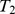 on taxa set 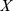 is defined as 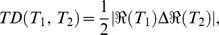
where 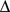 is the symmetric difference between two sets. For example, for the two MUL trees 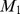 and 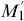 shown in Figure 7a and Figure 7b respectively, 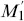 is consistent with all triplets in 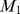 and has less duplication than 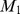. Since 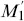 satisfies an extra triplet 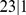 which is not contained in 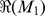, so 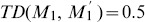. It shows that it is possible to present an algorithm satisfying all conditions of SMRT problem but does not return the correct MUL tree, that is, it does not satisfy the consistency principle of phylogeny reconstruction algorithms. Now, consider another two examples: MUL trees 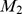 and 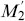 shown in Figure 7c and Figure 7d respectively. These MUL trees have the same number of duplications and 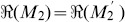, that is, 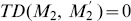. But these are different MUL trees because they have different duplication leaves and have different clusters. This situation happened because in a MUL tree, a triplet may occur several times. For example, the triplet 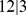 occurred three times in the MUL tree shown in Figure 8. This phenomenon exactly occurred in Figure 7c and Figure 7d. For instance, the triplet 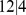 occurred in 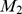 once whereas it occurred twice in 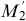. Hence, the rooted triplet distance introduced in Def. 1 does not properly show the distance between two MUL trees.

A multiset is defined as a 2-tuple 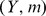 where 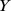 is some set and 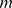 is a function from 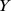 to the positive natural numbers 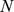. The set 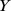 is called the underlying set of elements. For each 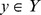, the multiplicity 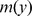 is denoted to be the number of occurrences of 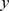. The symmetric difference between two multisets 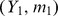 and 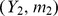 is denoted by 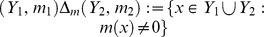, where 
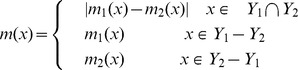



We also define the size of a multiset 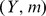 as 
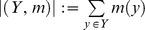
. For example, consider two multisets 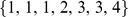 and 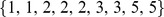. The symmetric difference between these sets and its size are 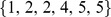 and 6 respectively. For a MUL tree 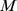, let 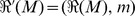 be the triplet encoding multiset of 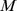. It means that if a triplet is seen in the MUL tree 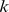 times, then 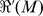 contains this triplet 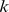 times. We define the new triplet distance between two MUL trees as follows:

#### Definition 2

- The rooted triplet distance between two rooted phylogenetic MUL trees 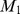 and 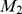 on taxa set 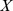 is defined as

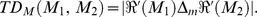

- The rooted triplet distance between a rooted phylogenetic MUL tree 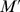 and a multiset of triplets 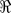 on taxa set 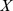 is defined as

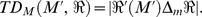

- The rooted triplet distance between two multisets of triplets 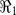 and 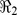 on taxa set 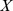 is defined as

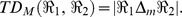

**Figure 9:**
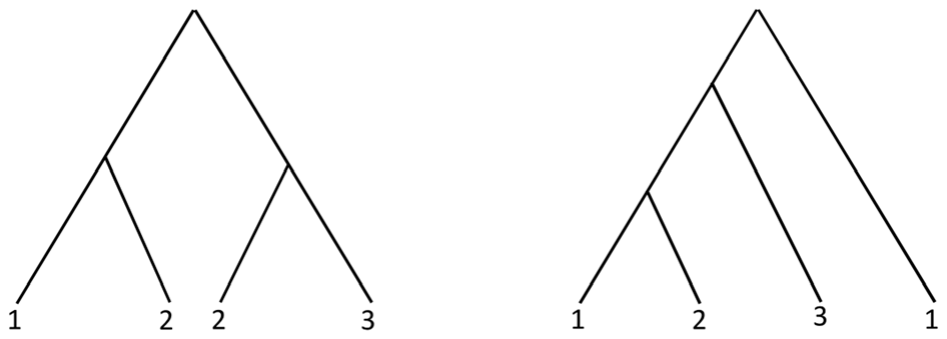
Two different MUL trees with tha same multiset of triplets 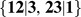.

Using the new rooted triplet distance 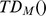 defined in Def. 2, the distance between MUL trees 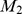 and 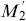 shown in Figure 7 equals 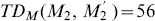. Note that a MUL tree is not uniquely defined by its multiset of triplets. For example, two MUL trees shown in Figure 9 have the same multiset of triplets. However, it seems that for most of the MUL trees specially for large MUL trees, it is true that two MUL trees are isomorphic if they have new triplet distance 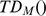 equal to 0. To show this, we computed the triplet distance 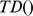 and new triplet distance 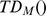 for all simulated and real datasets. The results of simulated datasets are shown in Table 1. Suppose 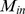 is a MUL tree and 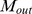 is the result of applying MTRT algorithm on 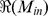. We define 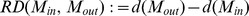. We classify the simulated datasets into 5 classes: 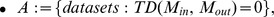


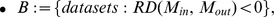


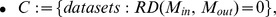


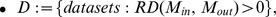


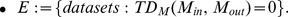



**Table 1:** The results of MTRT algorithm on simulated datasets.

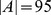	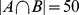	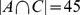	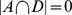	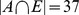
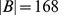	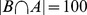	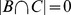	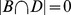	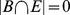
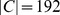	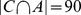	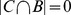	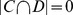	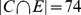
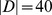	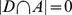	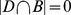	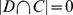	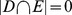
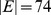	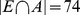	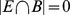	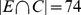	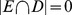

In the table, 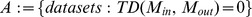, 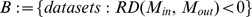, 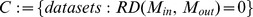, 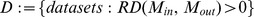 and 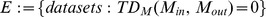 where 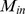 is a MUL tree and 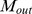 is the result of applying MTRT algorithm on 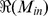.

Table 1 shows the intersection of above sets. For example, in 100 datasets, MTRT produces a MUL tree which has less duplication than that of the input MUL tree and the corresponding triplet distance is 0. In 74 datasets, the output and input MUL trees have the same number of duplications and the new distance between them is 0. We studied these 74 datasets and found that their corresponding output and input MUL trees are exactly the same. We also examined the exact algorithm on 100 datasets mentioned in Results section. The results show that in 56 datasets, the exact algorithm produces MUL trees which have less number of duplications than that of the original MUL tree. For the remaining datasets, the number of duplications for both MUL trees are the same. This shows that for more than fifty percent of the cases, the MUL tree produced by the exact algorithm is different from the input MUL tree. We also obtained the 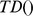 and 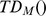 for real datasets. For the first real data, 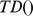 is 98, that is, the output MUL tree has 196 triplets which are not contained in input triplet set. 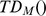 for this data is 2573. For second real data, 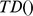 is 76.5, that is, the output MUL tree has 153 triplets which are not contained in input triplet set. 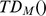 for this data is 6151. For third data, 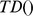 and 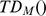 are 2 and 255 respectively. These numbers and Table 1 show that in many cases the SMRT problem and its conditions do not satisfy the consistency principle. Hence in many cases, the algorithms based on SMRT fail to produce the exact MUL tree.

## Discussion and Future Works

In this paper, we presented a heuristic algorithm MTRT for the SMRT problem. MTRT is implemented in MATLAB and is available at http://bs.ipm.ir/softwares/MTRT/. The goal of the algorithm is to construct a minimal MUL tree that is consistent with the input set of triplets and minimizes the number of its duplications. Note that a phylogenetic network can be associated to a MUL tree [14]. Therefore, it seems that constructing the smallest MUL tree from a set of triplets could be an alternative method for the problem of constructing a phylogenetic network with minimum reticulation from a set of triplets. To test the performance of the MTRT, we applied it on 400 simulated MUL trees and three real datasets. For each simulated and real MUL tree, we extracted all its triplets and applied the MTRT algorithm on the triplet set. We have shown that in most cases, the MTRT works well and has an acceptable running time. In only 10 percent of the datasets, the number of duplications for the output MUL tree of MTRT is greater than that of the original MUL tree. We also compared MTRT with exact algorithm. To do this, we executed the exact algorithm on 100 datasets. We showed that, in 86 datasets, the MUL trees produced by both MTRT and exact algorithm have the same duplications. We found that for more than 50 percent of the cases, the exact algorithm produces an output which is different from the input. It shows that the SMRT problem does not satisfy the consistency principle. So, having the set of triplets consistent to a MUL tree is not enough to infer that MUL tree. Furthermore, considering the minimum number of duplications to reconstruct a MUL tree that is consistent with a given set of triplets is not appropriate to infer the correct MUL tree. Therefore, from a biological point of view, there is a deficiency in the SMRT problem. Equivalently, the problem of constructing a phylogenetic network with minimum reticulation from a set of triplets is not consistent with the consistency principle of phylogeny reconstruction methods. It is necessary to consider other conditions to obtain proper MUL trees or phylogenetic networks. We extended the definition of triplet distance 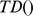 and introduced a new triplet distance 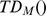. For all datasets, we compared the output MUL tree with original MUL tree by 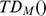. For all datasets with 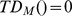, we showed that the output and original MUL trees are the same. According to these observations, we propose the following problem, called MUL tree from a multiset of rooted triplets with minimum triplet distance, or mMTd for short:

### 

#### mMTd problem

Given a multiset 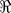 of rooted triplets over a leaf label set 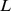, output a MUL tree 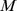 which minimizes 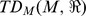.

Note that the maximum rooted triplets consistency problem, or MRTC for short [4], is a special case of mMTd problem. A natural question is how a multiset can be generated from biological data? For example, in the study of area cladograms, suppose a set of triplets is produced and we are interested to replace organisms by area names. Or in the other field, suppose we want to replace parasites by their host. Thus, a multiset of triplets may be derived from a great variety of biological processes.

We can simply extend the definition of the new triplet distance to a phylogenetic network. Hence, the other problem can be defined as follows, called Network from a multiset of rooted triplets with minimum triplet distance, or nMTd for short:

#### nMTd problem

Given a multiset 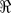 of rooted triplets over a leaf label set 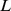, output a network 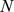 which minimizes 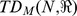.

## Materials and Methods

This section describes a heuristic method MTRT that aims to solve the SMRT problem. We first define the concept of a separating set in a graph. Consider a graph 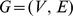. The subgraph 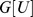 induced by 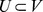 has a vertex set 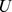 and an induced edge set 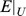 that consists of all edges in 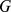 whose both endpoints lie in 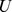. Suppose 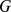 is a connected graph. The set 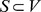 is called a separator, or a separating set, of 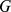 if 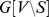 is disconnected. Now, let 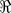 denotes a given set of triplets over a leaf label set 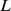. MTRT tries to build a MUL tree 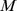 which is consistent with 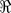 and its leaf duplications 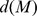 is as small as possible. MTRT is based on Aho et al.'s algorithm [1]. The Auxiliary graph, denoted by 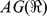, is required, which is a graph corresponding to 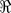 with vertex set 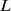 and edge set 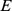 such that: 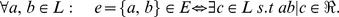



In general, the algorithm MTRT does the following steps. 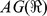 is computed first. If 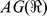 is disconnected, then the set 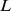 is partitioned into two non-empty sets 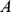 and 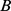 such that the set of vertices in each connected component of 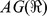 is a subset of either 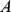 or 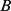. Now, the triplet sets 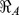 and 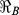 are computed. We set 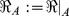 and 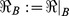. If 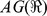 is connected, then MTRT tries to find the minimum separating set 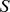 and classifies the connected components of 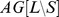 into two non-empty sets 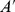 and 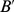. It is well known that finding the all minimum-size separators is an NP-hard problem [3]. To find a minimum separator, we use AllMinSep algorithm [2]. AllMinSep computes the set of all minimal separators of a graph G in time 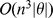 where 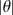 is the number of all minimal separators. AllMinSep first produces an initial set of minimal separators 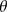. Then for each 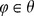, a family of other minimal separators is generated and added to 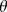. This procedure is done until all minimal separators are obtained, see [2] for more details. Since the number of all minimal separators can be exponential and we do not need all the minimal separators, so we use the AllMinSep with a small change to make it a greedy algorithm. Suppose the initial set of minimal separators 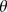 has been obtained and 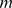 is the size of the smallest separator in 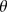. Then for each 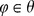, a family of other minimal separators 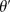 is generated. Now, the separator 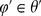 is added to 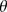 if 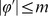.

Let 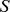 be a separator computed by AllMinSep and the connected components of 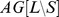 are classified in two non-empty sets 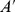 and 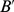. We set 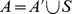 and 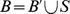. The triplet sets corresponding to 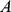 and 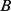 are considered as follows: 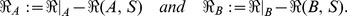



Now, the algorithm recursively handles sets 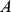 and 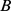 with triplet sets 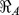 and 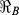 respectively. Let the MUL trees constructed by MTRT for the sets 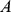 and 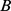 are 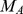 and 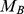 respectively. We report the MUL tree 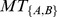 formed by connect 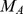 and 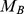 with the same root. For the case that 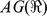 is connected, we define 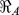 and 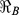 in such a way because the members of 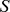 are repeated on both sides of the root. So, the set 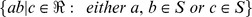 is consistent with the 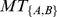 and it is unnecessary to consider this set. It is obvious that the output MUL tree of the algorithm is consistent with 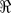. We now illustrate the steps of the algorithm MTRT by an example.

**Figure 10:**
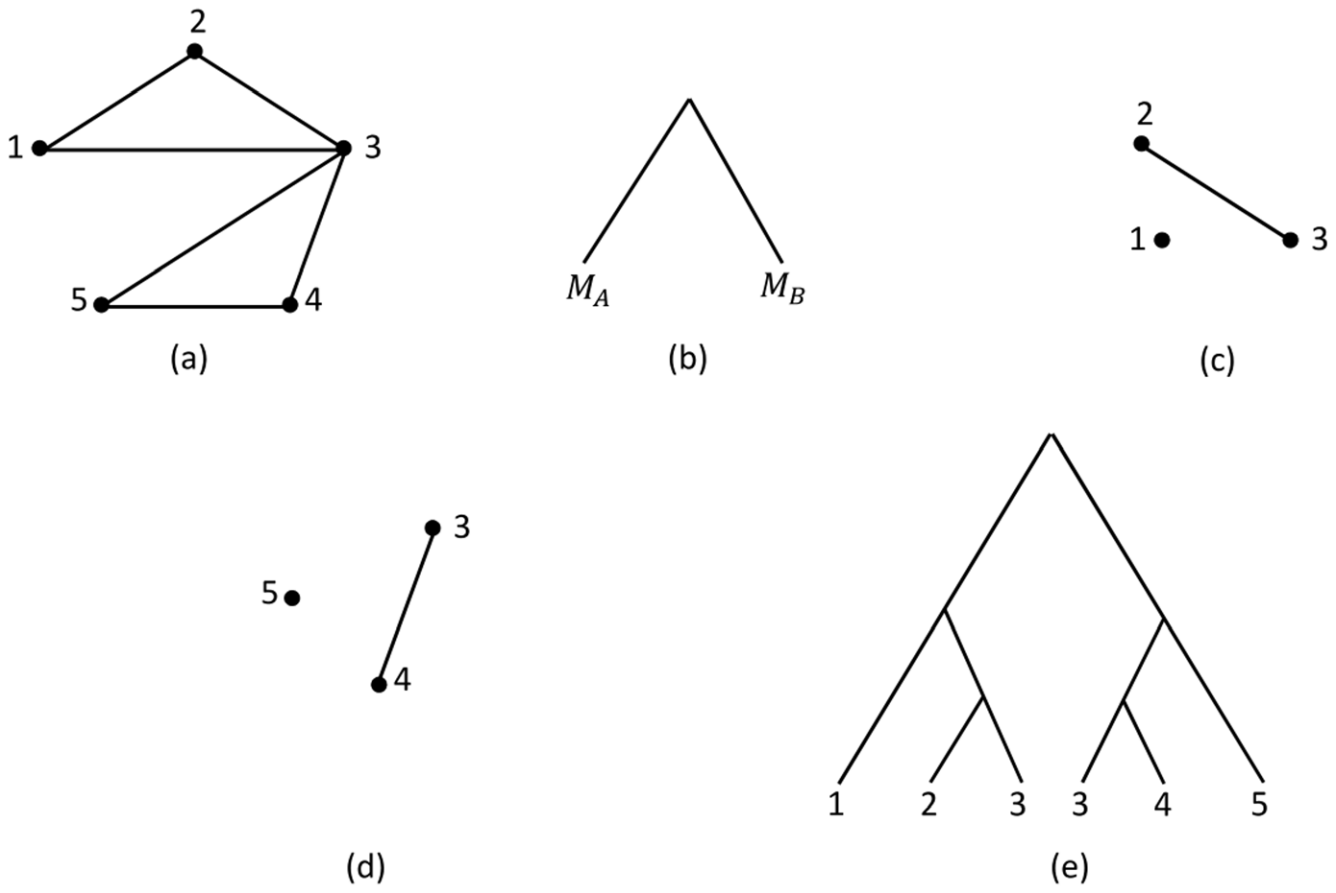
Steps of MTRT. (a) The auxiliary graph corresponding to 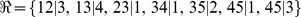, (b) 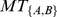, (c) The auxiliary graph 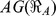, (d) The auxiliary graph 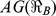, (e) A smallest MUL tree produced by MTRT algorithm.

Let 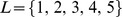 and 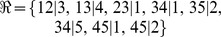 be the set of triplets over 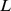. The auxiliary graph corresponding to 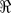 is shown in Figure 10a. The set 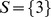 is the minimum separator of 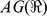. Hence, 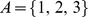 and 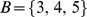. 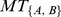 is shown in Figure 10b. The induced triplet sets for 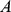 and 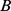 are 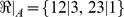 and 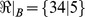 respectively. Now, 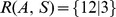 is removed from 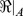 to obtain 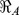. So, 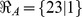 and 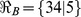. The auxiliary graphs 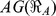 and 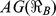 are shown in Figure 10c and Figure 10d respectively. Finally, the MUL tree produced by MTRT algorithm is shown in Figure 10e.

We now describe two cases that may occur in some steps of the algorithm:

### 

#### Case 1

It is possible at some steps of the algorithm, for a leaf label set 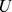, 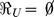. In this case, the triplets of an arbitrary tree on 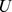 is considered as 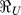. For instance, let 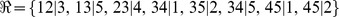. The separator of 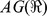 is 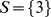. So, 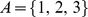, 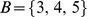, 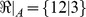 and 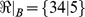 and consequently, 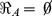 and 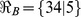. Now, an arbitrary triplet set consistent with a tree on leaf label set 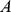 is considered as 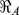, for example 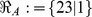. If the algorithm runs to the end, the MUL tree shown in Figure 10e is produced.

#### Case 2

There are more than one minimum separating set. In this case, MTRT chooses a separator 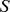 with minimum 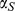, where 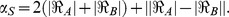



**Figure 11:**
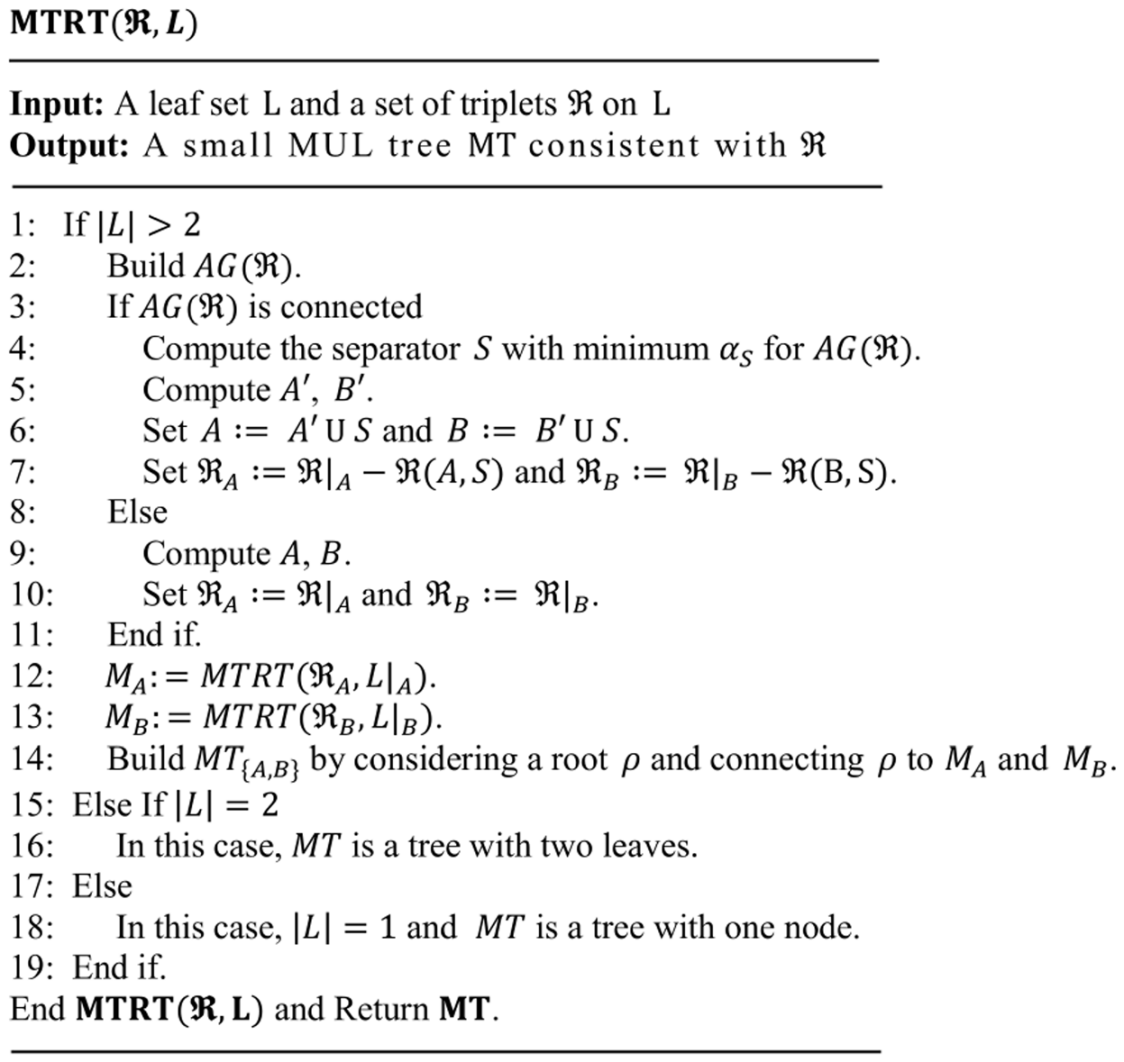
Pseudocode of the MTRT algorithm.

If 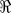 has more triplets, then the probability of having more duplications is high. The first part of 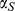 help to reduce the number of duplications and the second part of 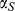 help to produce a MUL tree which is relatively balanced. Since minimizing the number of triplets is more important, we give bigger weight (2, by default) for the first part. The pseudocode of the MTRT algorithm is detailed in Figure 11.
